# Artificial Intelligence in Gynecological Oncology from Diagnosis to Surgery

**DOI:** 10.3390/cancers17071060

**Published:** 2025-03-21

**Authors:** Stefano Restaino, Maria Rita De Giorgio, Giulia Pellecchia, Martina Arcieri, Francesca Maria Vasta, Camilla Fedele, Paolo Bonome, Giuseppe Vizzielli, Sandro Pignata, Gaia Giannone

**Affiliations:** 1 Clinic of Obstetrics and Gynecology, “Santa Maria della Misericordia” University Hospital, Azienda Sanitaria Universitaria Friuli Centrale, 33100 Udine, Italy; pellecchia.giulia001@spes.uniud.it (G.P.); martina.arcieri@asufc.sanita.fvg.it (M.A.); giuseppe.vizzielli@uniud.it (G.V.); 2 PhD School in Biomedical Sciences, Gender Medicine, Child and Women Health, University of Sassari, 07100 Sassari, Italy; 3 U.O.S.V.D. Oncological Screenings, “Vito Fazzi” Hospital, 73100 Lecce, Italy; mariarita.degiorgio@asl.lecce.it; 4 Department of Medicine, University of Udine, 33100 Udine, Italy; 5 Department of Obstetrics and Gynecology, IRCCS Ospedale San Raffaele, 20132 Milano, Italy; vasta.francescamaria@hsr.it; 6 UOC Ginecologia Oncologica, Dipartimento di Scienze Della Salute Della Donna, Del Bambino e di Sanità Pubblica, Fondazione Policlinico Universitario A. Gemelli, IRCCS, 00168 Rome, Italy; camilla.fedele@guest.policlinicogemelli.it; 7 Radiation Oncology Unit, Gemelli Molise, Responsible Research Hospital, 86100 Campobasso, Italy; paolo.bonome@libero.it; 8 Oncologia Clinica Sperimentale Uroginecologica, Istituto Nazionale Tumori IRCCS Fondazione G. Pascale, 80131 Naples, Italy; s.pignata@istitutotumori.na.it; 9 Ovarian Cancer Action Research Centre, Department of Surgery and Cancer, Imperial College London, London SW7 2AZ, UK

**Keywords:** artificial intelligence, gynecology oncology, surgery, diagnosis

## Abstract

**Background:** The field of medicine, both clinical and surgical, has recently been overwhelmed by artificial intelligence technology, which promises countless application scenarios and, above all, implementation in clinical practice and research. Novelties are riding the wave fast, but where do we stand? A small overview in gynecological oncology of future challenges, evidence already investigated, and possible scenarios to be derived was conducted. **Methods**: Both diagnostic and surgical work in the field of gynecological oncology was conducted, selecting the most interesting articles on the subject. **Results**: From the narrative review of the literature, it emerged how much further ahead the diagnostic field is at present compared to the surgical one, which appeared to be more limited to ovarian surgery. Most current evidence focuses on the role of different biomarkers in predicting diagnostic, prognostic, and treatment-integrated patterns. **Conclusions**: Everything we know to date is related to a dynamic photograph that is constantly and rapidly changing as much as AI is becoming inextricably linked to our medical field.

## Simple Summary

Artificial intelligence (AI) is revolutionizing modern medicine, particularly in the field of gynecological oncology, where its applications extend across diagnostics, prognostics, and treatment planning. This comprehensive overview explores the latest advancements and persistent. challenges in integrating AI into both clinical and surgical practices. In diagnostics, AI-driven innovations, particularly biomarker-based approaches, have significantly enhanced early detection and personalized treatment strategies. However, despite these advancements, the adoption of AI in surgical applications, such as ovarian cancer surgery, has been comparatively slower. This disparity highlights the need for further research, validation, and integration of AI-driven tools into surgical workflows to optimize precision, patient outcomes, and overall clinical decision-making in gynecological oncology.

## 1. Introduction 

The application of machine learning (ML) and artificial intelligence (AI) approaches to the study of gynecologic cancers has shown potential to revolutionize cancer detection; some experiences have been published in the area of oncological surgery. AI-based research around biomolecular screening and diagnostics of gynecologic cancers has increased dramatically in the last five to ten years [1]. In particular, AI is playing an important role in areas of medical research, adding the ability to extract a huge quantity of computer-derived measurements from digitalized data. Applications include imaging with path-omics, genetic diagnosis with genomics, and transcriptomics, enzyme assessment with proteomics and metabolomics, as well as improving detection and personalization in the setting of radiomics [2]. AI nowadays takes the form of countless declinations of meaning. In gynecologic oncology, it translates into both the definition of predictive diagnostic, prognostic, training, and information or misinformation models. In many diagnostic medical settings, deep learning (DL) models are demonstrating great capacity for improvement in clinical practice. AI is amazing in its ability to learn from the many inputs it can receive and merge [3]. In gynecologic oncology, the same strand is shared: a predictive ultrasound model of differential diagnosis between atypical hyperplasia and endometrial carcinoma was published and is the epitome of it [4]. Nevertheless, technical and clinical issues hamper the application of AI in gynecological oncology. One of them, probably the most crucial, is automation bias: some AI techniques lack transparency in their decision-making processes, and their applicability and generalizability are also affected by the size of the sample to which they can be applied. The method of reaching certain conclusions can be difficult to understand, and this does not mesh well with the universality of the scientific rationale [5]. As evidence of this, a PubMed search for articles on artificial intelligence and gynecological–oncological surgery yields very few results, in stark contrast to the extensive presence of machine learning research in other areas. In this narrative review, we aim to provide an overview of the major advances and future perspectives on the use of AI in the setting of gynecologic oncology for diagnostics and surgery. Our intention is to provide a summary of what evidence has been supplied to date on the application of AI in gynecologic oncology, also taking into account the future scenarios at stake, which ones can be improved, and which others may open up.

## 2. Materials and Methods

For the purpose of this literature review, AI has been used as a MESH term on PubMed and associated with terms such as “gynecologic cancer” or each single tumor, such as “ovarian cancer”, “endometrial cancer”, or “cervical cancer”, updated to the 24 August 2024. Roughly summarizing, though the vast majority of publications in the area of screening and diagnostics are related to cervical cancer (852 publications, last PubMed search on 24 August 2024, mostly related to cytology vs. 600 publications for ovarian cancer and 353 for endometrial cancer) or to imaging technology, when it comes to the possibility of the early detection of the molecular signature of cancer thanks to AI, most of the research efforts around the world are focused on ovarian cancer. For the surgical counterpart, MESH terms such as surgery and AI and gynecology oncology have been in various ways, and in the form of synonyms, applied to research, though not systematically, since this is a narrative work. We selected published evidence about the role of AI in gynecological oncology diagnosis and surgery and summarized the main findings. Topics of interests were applications of AI in supporting and improving diagnostic and surgical performance in the field of interest. In parallel, work focusing on pertinent reviews that were informative about the application of AI in the medical scientific field, its pitfalls, and strengths in clinical practice was also of interest to us (Supplementary Table S1).

## 3. Results

For diagnostic purposes, the vast majority of the publications in the area of screening and diagnostics were related to cervical cancer. There are a lot fewer studies about AI’s application in the field of gynecological tumor surgery; studies appear to only be currently available for uterine and ovarian cancers.

### 3.1. Diagnostic Perspectives

AI applications range from a re-evaluation on a predictive model of already known biomarkers to the study of specific metabolites, as demonstrated by the studies we are going to describe below. AI has been used to perform risk assessments of adnexal masses. In 2022, Reilly G. and colleagues proposed an AI model named MIA3G, a deep feedforward neural network for ovarian cancer (OC) risk assessment, designed to use seven protein biomarkers along with age and menopausal status as input features. MIA3G was developed on a heterogenous dataset of 1067 serum specimens from women with adnexal masses and subsequently validated on a cohort almost twice that size. The seven biomarkers used in the MIA3G algorithm are CA125, HE4, beta-2 microglobulin, apolipoprotein A-1, transferrin, transthyretin, and follicle-stimulating hormone. Overall, in the analytical validity dataset, which simulated real-world prevalence for ovarian malignancy (4.9%), the algorithm demonstrated a sensitivity of 89.8%, a specificity of 84.0%, a positive predictive value of 22.5%, and a negative predictive value of 99.5%. The training and testing stage used 1,050 specimens with 30% positive specimens, indicative of a high-risk OC population. This development was followed by a detailed validation process on 2000 specimens that show performance in a low-prevalence population (approximately 5%), making the results promising, albeit the retrospective nature of the dataset and the histologic subtypes imbalance [6]. More recently, a multicenter retrospective study was published, in which 52 features from 99 items (98 regular laboratory tests and age, starting from 6,778,762 laboratory examinations from three large hospitals in China) were screened to build the multi-criteria decision-making-based classification fusion (MCF) model, which integrated the best 20 artificial intelligence classification models, for the accurate identification of patients with a high risk of ovarian cancer in an Asian population. The MCF model showed high prediction efficacy in differentiating individuals with OC versus those who did not have OC, with an AUC of 0.949 (CI 95% 0.948–0.950), outperforming CA125 and HE4 alone in identifying ovarian cancer, especially in early-stage ovarian cancer prediction [7]. Abrego et al., using data from the UK Collaborative Trial of Ovarian Cancer Screening, also tested different combinations of biomarkers, including CA125, for the prediction of OC using recurrent neural networks (RNNs) with longitudinal observations. Though encouraging in the design, the study was limited by a small sample size and the need for a larger panel of biomarkers [8]. Another application of AI in the diagnostic setting has been to increase the detection rate of available diagnostic tests, such as cancer antigen 125 (Ca125). Specifically, LeBien and colleagues in their study have explored the possibility of estimating accurate age-specific reference intervals (RIs) for CA-125 in a Puerto Rican cohort of women, using a convolutional neural network (CNN). Using two distinct indirect methods, they predicted a similar, highly variable pattern of CA-125 reference intervals across different age groups, suggesting that the widely accepted upper limit of CA-125 of 35 U/mL should not be applied universally, but age-adjusted cut-offs should be developed and validated [9]. The use of AI moved beyond the available test to improve the detection rate of tools used in research. As an example, it has been applied on genome-wide methylation analysis of cfDNA and plasma cfDNA fragmentomics, with the development of the DNA Evaluation of Fragments for Early Interception (DELFI), which assesses fragment coverage, size, and other summary statistics within 5 Mb windows [10,11,12,13]. In other studies, ML has been applied in efforts to identify patterns embedded within metabolomics, since perturbations of metabolite levels in the blood and/or other body fluids constitute a molecular phenotype reflective of underlying collective information encoded at the genome level and realized at the transcriptome and proteome levels. As such, metabolic profiles have long been considered promising indicators of cancer and other complex diseases [14]. Ban and colleagues, in a work published in 2024, aimed to develop an ML-based approach for predicting OC using metabolomic profiles (3-Hydroxydodecanedioic acid, ceramide) in serum samples collected from 431 OC patients and 133 healthy donors (showing a positive predictive value (PPV) of 93%) [15]. Another study published in 2023 used multiple metabolites associated with OC and involved in five metabolic pathways linked to OC: Nicotinate and Nicotinamide Metabolism, Glycolysis/Gluconeogenesis, Aminoacyl-tRNA Biosynthesis, Valine, Leucine and Isoleucine Biosynthesis, and Alanine, Aspartate and Glutamate Metabolism. Several classification models for the identification of OC using related metabolites were created and their accuracies were confirmed through testing with 10-fold cross-validation. In this study, the most accurate model was able to achieve 85.29% accuracy, further suggesting the possibility of developing machine learning models for OC diagnostics using metabolomics data [16]. AI has been applied to both cfDNA and metabolomics to also risk-stratify women with uterine masses. Low-coverage whole-genome sequencing and an extensive set of genome-wide features derived from cfDNA fragmentomics have been used to differentiate between uterine corpus endometrial carcinoma (UCEC) and healthy conditions, and the results were evaluated using an AI-based algorithm. In total, 111 UCEC patients and 111 healthy donors were analyzed using an ML framework which included three distinct features: copy number variations (CNVs), feature selection dimensionality (FSD), or nuclear features (NFs). The model demonstrated excellent predictive power, as evidenced by high AUC values in both the training and independent validation cohorts [17]. Blood metabolome was analyzed: metabolites were extracted from dry blood samples of all participants and analyzed by gas chromatography–mass spectrometry. This has been evaluated in postmenopausal women not receiving hormonal therapy showing a greater than 99% accuracy in predicting the presence of EC. The diagnostic study enrolled two cohorts, a multicenter prospective cohort, with 50 cases (postmenopausal women with EC; FIGO stage I–III and grade G1–G3) and 70 controls (no EC but matched on age, years from menopause, tobacco use, and comorbidities), used to train multiple classification models. The accuracy of each trained model was then used as a statistical weight to produce an ensemble ML algorithm for testing, which was validated with a subsequent prospective cohort of 1430 postmenopausal women. The overall screening test showed no false-negative results and two false-positive results based on 1430 analyzed samples. The accuracy was 99.86% [18].

### 3.2. The Role of AI in the Setting of Gynecologic Oncology Surgery

Other significant work, which has benefits that can be generalized to gynecological oncology surgery as a whole, has applied machine learning models like extreme gradient boosting, Random Forest, and Logistic Regression to assess the risk of residual disease post-hysterectomy for gynecological oncological conditions, based on clinical and surgical parameters. AI models revealed that the top postoperative predictors of residual disease were the initial presence of gross abdominal disease on the diaphragm, disease located on the bowel mesentery, located on the bowel serosa, and disease located within the adjacent pelvis prior to resection. No significant difference between the models was found. Models contributed to enhancing clinical choice for adjuvant treatment [19].

Not only in the treatment setting but also on perioperative and mortality outcomes may AI find applications.

The PROMEGO (Predicting Risk of Post-Operative Morbidity and Mortality following Gynaecological Oncology Surgery) study focused on evaluating the risk of postoperative morbidity and mortality. Utilizing the international GO SOAR database dataset, authors have developed a novel predictive surgical risk calculator for postoperative morbidity and mortality following gynecological surgery [20]. This is a novel tool, promising to be very useful for surgeons in the definition of cytoreductive surgery. Preliminary data showed accurate prediction of thirty-day postoperative morbidity by using variables readily available across all resource settings [21].

### 3.3. Application of AI in Ovarian Cancer Surgery

The greatest interest in AI in surgery is ovarian cancer, where it represents a challenging and surprising tool in the hands of surgeons. High-grade serous ovarian cancer (HGSOC), the most frequent and one of the most aggressive epithelial histotypes, is targeted by AI models in an attempt to define predictive algorithms in pre-, intra-, and postoperative care settings. “Open & close” surgeries are one of the main issues in the planning treatment of patients with HGSOC, who often only undergo a simple surgical exploration, instead of the planned HIPEC plus cytoreduction with radical intent, because of the intraoperative unexpected finding of unresectable disease. In this regard, Maubert et al. developed prediction algorithms by comparing various machine learning models to determine if one was superior in terms of effectiveness and accuracy. They identified intestinal and pelvic carcinosis as the main criteria of non-resectability out of nine, using a combination of clinical and imaging data [22].

A predictive ML/deep learning (DL) score—the Leeds L-AI-OS Score—to predict the length of stay after cytoreductive surgery emerged as effective in evaluating simple preoperative variables such as age, BMI, and ECOG PS, intraoperative time, surgical complexity score (SCS), and estimated blood loss [23]. In a similar patient context, utilizing comparable variables along with the inclusion of intestinal resection with ostomy, the Graphical User Interface Calculator established the Leeds Natal Score. This score accurately predicts the risk of ICU admission, helping to optimize ICU places and plan the surgical schedule effectively [24]. In the early days, quantitatively predictive factors of ‘surgical effort’ also emerged. Notably, XGBoost (extreme gradient boosting) and DNN (Deep Neural Network) AI models enabled the establishment of an SCS cut-off of 5, above which the probability of ineffective cytoreduction increases [25]. The ANAFI score, utilizing the same XGBoost model, was developed to intraoperatively predict the likelihood of achieving complete cytoreduction when disease has specific anatomical fingerprints: small bowel mesentery, large bowel serosa, and diaphragmatic peritoneum disease [26]. The same score also indicated that it was the main prognostic feature for survival outcomes. This appears as an innovative feature as AI scores are, in general, mostly focused on the prediction of suboptimal surgery [27].

Laios et al. instead, with datasets from ESGO-accredited centers for surgery in ovarian cancer, identified upper abdominal peritonectomy (UAP) and regional lymphadenectomies as the main features predictable of complete cytoreduction [28].

Furthermore, the role of human epididymis protein 4 (HE4) was studied, a tumor biomarker identified together with CA125 antigen useful to estimate the risk of identifying malignant ovarian cancer in surgery. Its preoperative level could help to identify patients at higher risk for suboptimal cytoreductive surgery or those who may require more extensive surgery [29].

Further prospective studies are needed to explore the prognostic utility of combining clinical, radiological, and biological parameters, particularly through the use of artificial intelligence-based models.

AI has been developed also in the recurrent setting of ovarian cancer. Bogani et al. in 2018 used artificial neuronal network (ANN) to search for variables correlating with secondary cytoreductive surgery (SCC) and complete resection with no residual macroscopic disease in 194 patients with platinum sensitive recurrent ovarian cancer, candidates for surgical resection [30]. They found three main factors driving the ability to achieve it: disease-free interval (DFI), considered as the time between the end of platinum-based adjuvant chemotherapy and the diagnosis of recurrence; retroperitoneal recurrence; and FIGO stage at diagnosis. They were quantitatively pooled: DFI was the most important factor influencing overall survival (OS) [30]. Three outcomes were considered: the chemotherapy response score (CRS) on omentum, residual disease after IDS, and recurrence itself.

### 3.4. AI Premises for Uterine Cancers Surgeries

The aid of artificial intelligence in the treatment of uterine neoplasms has currently found less room for study. One of the hottest topics at the moment, and one in which AI could well establish itself as a supporting tool, is the correlation between atypical endometrial hyperplasia and the existing presence of endometrial carcinoma. One such work has just been published, which compared different artificial intelligence models of varying degrees of complexity and accuracy and different variables. From the results, it emerged that no model achieved a sensitivity greater than 50%, concluding that currently it is not possible to predict concurrent EC in women with a preoperative diagnosis of EIN [31]. Similar findings had already emerged a few months back from a multicenter retrospective Italian study [32]. In contrast, the pursuit of defining a unique metabolic signature for endometrial cancers appears to be more promising [33]. This study investigated the potential of metabolomics as a non-invasive and reliable screening tool for endometrial cancer (EC), aiming to validate the efficacy of metabolomic profiling in detecting EC by analyzing a large cohort of women undergoing gynecological surgery (for EC versus for benign conditions). Several metabolites, particularly those involved in lipid and amino acid metabolism, were identified as potential biomarkers potentially facilitating earlier tumor diagnosis with a formidable impact in tailored therapeutic strategies [33]. Regarding cervical cancers, the application of AI in the prediction of parametric infiltration in early-stage cervical cancers in the era of the SHAPE trial revolution emerges as the first publication [34]. iPMI, an algorithm model proposed in this regard in a recent article published in *Cancers*, could serve as a cost-effective and rapid approach to guide important clinical decision-making [35]. Under investigation is finally a deep learning model of survival to predict the prognosis of operable cervical cancer patients [36]. Preliminary data are encouraging.

## 4. Discussion

### Future Perspectives

As Moro et al. demonstrated, the role of AI in clinical management could be very significant [37]. For diagnostic application, first, it has the potential to improve diagnostic accuracy, which could reduce the time clinicians spend on diagnosis. Additionally, it could allow more time for clinicians to focus on the interpersonal aspects of patient care. Ultimately, a key AI application in ultrasound for gynecologic oncology is enhancing the accuracy of preoperative diagnoses for ovarian masses, supporting clinicians and surgeons in treatment planning, even in the absence of expert ultrasound technicians [37]. Certainly, many surgical fields await to be investigated, and at the moment, for the vulva, there are no studies available. AI can play a role of upmost importance that perhaps should be investigated more than any other: implementing and speeding up the training of young clinicians, allowing for faster learning times and improving technical skills [38]. Undoubtedly, this is a highly relevant area of research, as evidenced by the wide range of scientific publications covering various aspects, including screening, diagnostics, surgery, histology, oncology, and prognostic assessment. These developments highlight the growing impact of AI across multiple facets of gynecological oncology [39,40,41,42,43,44]. AI is set to transform both research and clinical practice in gynecological oncology in numerous ways. Current clinical trials, as summarized in Supplementary Table S2, highlight key areas where AI is being applied, including palliative care, genetic syndrome prevention, immunotherapy response prediction, the histopathological prediction of tumors with uncertain malignancy significance, and radiomic diagnostic features for the optimization of the subsequently received treatment. These emerging themes illustrate AI’s potential to improve patient outcomes in various aspects of gynecological cancer care. These observations highlight an important consideration: emerging fields in gynecological oncology, such as immunotherapy, are closely tied to the integration of artificial intelligence. This suggests that advancements (of novel therapeutic approaches and any area of research that may be supported by AI-related tools. The development of such tools by the cooperation of researchers in the area of oncology and in the area of informatics may highly contribute to the future of cancer care) are interconnected and mutually dependent, with AI playing a crucial role in shaping the future of cancer care alongside new treatment approaches like immunotherapy. One does not exist without the other.

Notably, this integration of AI and emerging treatments may contribute to the development of precision medicine and transform cancer care into a more personalized and effective approach. The ongoing research and advancements will determine whether this synergy can deliver tailored treatments for each patient [45]. From our results, it became clear that much remains to be seen in terms of outcomes on surgical applications, and so much can still be pulled out on the diagnostic side. Table 1 lists the topics already discussed narratively, or systematically, on the applications of AI in gynecologic oncology, ranging from screening to treatment. Indeed, the gaps in Supplementary Table S3 show that further investigations are still needed.

Both deep learning and machine learning tools can detect computer system errors, either autonomously or with human oversight. A computer can identify errors through anomaly detection, while a physician may interpret model outputs to catch mistakes. However, automatic bias in AI systems can lead to incorrect detections if the training data are skewed or incomplete. Bias can arise from historical data patterns, reinforcing disparities and affecting decision-making, requiring careful monitoring and correction. Therefore, doctors should verify AI-generated results by cross-checking with clinical guidelines, patient history, and their own expertise. They should also use multiple AI tools for comparison and stay aware of potential biases in training data. Regularly auditing AI models, understanding their decision-making processes, and collaborating with data scientists can help mitigate errors. When in doubt, manual review and second opinions remain crucial.

Furthermore, the other side of the coin is not so idyllic because of the concerns that this fascinating and dark world both arouse and imply. Among them are the following:

- Legal Issues and Diagnostic Decisions Based on Deep Learning Without Expert Opinions: Deep learning models can assist in diagnostic decision-making, but they should not replace expert opinions entirely. Legal concerns arise if the AI model makes a wrong diagnosis, leading to liability issues. In most cases, medical decisions still require a professional’s oversight, and guidelines must consider the possibility of human error in AI applications.
- Deep Learning and Machine Learning Errors: Deep learning tools may be able to detect errors within their own algorithms (self-checking mechanisms), but the detection of computer system errors often requires intervention from either another machine or a physician. Automatic bias occurs when models are trained on biased datasets, which can lead to skewed predictions, potentially exacerbating health disparities.
- Approval Process for AI Tests or Methods: To set up an approval process, regulatory agencies (e.g., FDA in the U.S.) should assess AI tools for safety, efficacy, and transparency. This typically involves rigorous clinical trials, validation, and adherence to ethical standards. AI methods need to be tested for robustness, reproducibility, and alignment with medical guidelines.

The effort will have to be to always keep the use of AI subject to the guidance of the human mind experienced in the specific field to limit these implications.

## 5. Conclusions

AI algorithms require large datasets to build extensive databases, and these systems are developed and overseen by people. Furthermore, maintaining data quality and tracking over time requires trained and skilled personnel. In the future, the current landscape indicates that AI will have a significant impact on the diagnosis and treatment of gynecological tumors, as evidenced by several ongoing clinical trials.

With this in mind, we expect that in both the diagnostic and surgical fields with possible intersecting scenarios, we will soon have big news of new applications of increasingly personalized AI models to precision medicine of individual patients’ tumors. However, how this influence will be integrated into clinical practice remains to be discovered.

## Figures and Tables

**Table 1:** Topics discussed on AI applications in gynecologic oncology, from screening to treatment.

Title	Author	Year	Focus
Applying Artificial Intelligence to Gynecologic Oncology: A Review [46]	David Pierce Mysona et al.	2021	Overview of AI’s role in enhancing diagnosis, clinical decision-making, and personalized therapies in gynecologic cancers.
A Systematic Review on the Use of Artificial Intelligence in Gynecologic Cancer Imaging—Background, state of the art, and future directions [47]	Pallabi Shrestha et al.	2022	Discusses AI concepts and computer vision methods in the context of gynecologic cancer imaging.
Artificial Intelligence in Gynaecological Malignancies: Perspectives of a Clinical Oncologist [48]	Himanshi Khattar et al.	2023	Reviews AI’s role in various steps of the workflow of gynecological malignancies and discusses clinical aspects for future research.
Artificial Intelligence in Gynaecology Oncology [49]	Royal College of Obstetricians and Gynaecologists	2024	Explores the potential of AI to improve accuracy and efficiency in gynecological oncology diagnosis and treatment.
Artificial Intelligence in Women’s Health: A Comprehensive Review of Artificial Intelligence Advancements in Gynecology [50]	Marta Brandão et al.	2024	Aim to establish the current status of AI in gynecology, the upcoming developments in this area, and discuss the challenges facing its clinical implementation, namely the technological and ethical concerns for technology development, implementation, and accountability.

## Data Availability

No new data were created or analyzed in this study. Data sharing is not applicable to this article.

## Supplementary Materials

The following supporting information can be downloaded at: https://www.mdpi.com/article/10.3390/cancers17071060/s1, Table S1: Search strategy; Table S2: Selected ongoing trials for application of AI in gynecological oncology; Table S3: Gaps of the literature about AI in gynecology oncology.
