# Supplementary material for: Artificial Intelligence in Gynecological Oncology from Diagnosis to Surgery
Source: Cancers (Basel). 2025 Mar 21;17(7):1060. doi: 10.3390/cancers17071060 (PMC11987942; doi:10.3390/cancers17071060)
Supplement: Supplementary file 1 [file cancers-17-01060-s001.zip › cancers-3499143-supplementary.pdf]

Supplementary Materials

Supplementary Table S1. Search strategy.

Step	Details
**1. Initial Search**	AI as MESH term combined with "gynecologic cancer" and specific tumors (ovarian, endometrial, cervical cancer).
**2. Screening & Diagnostics**	**Cervical cancer:** 852 publications (mostly cytology-related).<br>**Ovarian cancer:** 600 publications.<br>**Endometrial cancer:** 353 publications.<br>**Imaging technology:** Major research focus.<br>**Molecular detection:** Primarily focused on ovarian cancer.
**3. Surgical Applications**	Research conducted with varied MESH terms (AI + surgery + gynecologic oncology). No systematic review due to narrative approach.

Supplementary Table S2. Selected ongoing trials for application of AI in gynecological oncology.

Clinical trial	Promotor	Title	Aim
NCT05709769	China	Radiomics-based Malnutrition for Cervical Cancer	To analyze the radiomics features of psoas extracted at the level of the third lumbar vertebra (L3) and then, develop a CT-based radiomics nomogram prediction model for predicting malnutrition based on their PG-SGA scores in patients with FIGO, 2014 stage IB1-IIA2 CC who received postoperative RT/CRT.
NCT05170087	Canada	Radiomic Analysis for Predicting Treatment Response and Clinical Outcomes in Malignancies	Development of imaging biomarkers in predicting treatment response for breast, brain, head-neck, and gynecological malignancies
NCT06279832	Italy	Radiomics and Radiogenomics Models to Predict Molecular Integrated Risk Classes and Prognostic Factors in Endometrial Cancer<br>ID: ROMANTIC STUDY	To develop radiogenomics models to stratify patients into three main risk categories (Favorable, Intermediate, and Unfavorable) according to the ProMisE model and use these models to predict the most prognostically relevant EC histopathological features.
NCT06182332	USA	An Artificial Intelligence Algorithm for Identifying Gynecologic Cancer Patients in Need of Outpatient Palliative Care	To test an AI algorithm that analyzes medical records to identify patients with advanced gynecologic cancer who may benefit from early palliative care consultation.
NCT05562778	USA	Chatbot to Maximize Hereditary Cancer Genetic Risk Assessment	To compare a mobile health platform, known as a 'chatbot,' that leverages artificial intelligence and natural language processing to scale communication, to 'usual care' that patients would receive.
NCT06540846	France	Deep Learning for Histopathological Classification and Prognostication of Gynaecologic STUMP	To develop a diagnostic and prognostic algorithm to help pathologists better classify and diagnose uterine smooth muscle tumors and predict their clinical course.
NCT05805358	Taiwan	Hyperpolarized 13C MRI for Cancer Immunotherapy	To investigate the use of hyperpolarized 13C MRI to assess effectiveness of immunotherapy
NCT04454450	USA	Using Advanced Imaging Studies to Develop a Profile of High-grade Serous Ovarian Cancer	To analyze the combined results of the imaging, genetic and immune system tests on the tumor samples of HGSOC
NCT04511481	China	Deep Learning Magnetic Resonance Imaging Radiomic Predict Platinum-sensitive in Patients With Epithelial Ovarian Cancer	To develop and validate EOC deep learning system

Legenda: PPV: positive predictive value; NPV: negative predictive value; RT/CHT: radiotherapy/chemoradiotherapy; PG-SGA: Patient-Generated Subjective Global Assessment; FIGO: International Federation of Gynecology and Obstetrics; CC: cervical cancer; STUMP: Smooth Muscle Tumours; HGSOC: high grade serous ovarian cancer; MRI: magnetic resonance imaging; EOC: epithelial ovarian cancer.

Supplementary Table S3. Gaps of the literature about AI in gynecology oncology.

Gap	Description
Limited AI Integration in Surgery	Most AI applications focus on diagnostics, with fewer studies on AI-assisted gynecologic oncology surgeries.
Small and Biased Datasets	Many AI models are trained on small, region-specific datasets, limiting generalizability and clinical application.
Lack of External Validation	Few studies validate AI models on independent, diverse patient populations.
Explainability and Transparency Issues	AI decision-making processes remain opaque, making clinical adoption challenging due to lack of trust.
Automation Bias in Decision-Making	Over-reliance on AI predictions without human oversight may lead to diagnostic errors.
Ethical and Legal Considerations	Unclear regulations on AI-driven diagnostics and treatments raise concerns about liability and patient safety.
Limited Real-World Clinical Implementation	Most research remains theoretical or in pilot phases, with few large-scale clinical trials evaluating AI efficacy.
Need for Interdisciplinary Collaboration	AI research in gynecologic oncology requires stronger collaboration between clinicians, data scientists, and regulatory bodies.
AI for Personalized Treatment	Few studies explore AI-driven personalized treatment strategies based on genomics and proteomics.
Long-Term Outcomes and Cost-Effectiveness	There is little research on the long-term impact and cost-benefit analysis of AI in gynecologic oncology.
